# Expression of Telomeric Repeat–Containing RNA Decreases in Sarcopenia and Increases after Exercise and Nutrition Intervention

**DOI:** 10.3390/nu12123766

**Published:** 2020-12-08

**Authors:** Ke-Vin Chang, Yu-Chen Chen, Wei-Ting Wu, Hong-Jhin Shen, Kuo-Chin Huang, Hsueh-Ping Chu, Der-Sheng Han

**Affiliations:** 1 Department of Physical Medicine and Rehabilitation, National Taiwan University Hospital, Bei-Hu Branch, Taipei 100, Taiwan; kvchang011@gmail.com (K.-V.C.); wwtaustin@yahoo.com.tw (W.-T.W.); 2 Community and Geriatric Medicine Research Center, National Taiwan University Hospital, Bei-Hu Branch, Taipei 108, Taiwan; bretthuang@ntu.edu.tw; 3 Department of Physical Medicine and Rehabilitation, National Taiwan University College of Medicine, Taipei 100, Taiwan; 4 Institute of Molecular and Cellular Biology, National Taiwan University, Taipei 106, Taiwan; r07b43028@ntu.edu.tw (Y.-C.C.); Owen11533@gmail.com (H.-J.S.); 5 Department of Family Medicine, National Taiwan University College of Medicine, Taipei 100, Taiwan; 6 Health Science and Wellness Center, National Taiwan University, Taipei 106, Taiwan

**Keywords:** sarcopenia, telomere, TERRA, exercise, nutrition

## Abstract

Sarcopenia is defined as aging-related loss of muscle mass and function. Telomere length in chromosomes shortens with age and is modulated by telomeric repeat-containing RNA (TERRA). This study aimed to explore the impact of aging and sarcopenia on telomere length and TERRA expression, and changes following strengthening exercise and nutrition intervention (supplement of branched-chain amino acids, calcium and vitamin D3) for 12 weeks in the sarcopenic population. Older adults (≥65 years old) were divided into non-sarcopenic controls (*n* = 36) and sarcopenic individuals (*n* = 36) after measurement of grip strength and body composition. The relative telomere length of leukocytes in all research participants was evaluated using the T/S ratio (telomere/single copy gene), and relative TERRA expression of leukocytes was determined by reverse-transcription qPCR (RT-qPCR). Generalized estimating equation (GEE) was used to analyze the influence of sarcopenia and intervention on the outcomes. There was no significant difference in telomere length between control subjects and participants with sarcopenia. TERRA expression was lower in sarcopenic participants compared to that in non-sarcopenic controls (5.18 ± 2.98 vs. 2.51 ± 1.89; *p* < 0.001). In the sarcopenic group, intervention significantly increased TERRA expression, but not telomere length. The GEE analysis demonstrated that TERRA expression was negatively associated with sarcopenia (β coefficient = −2.705, *p* < 0.001) but positively associated with intervention (β coefficient = 1.599, *p* = 0.023). Sarcopenia is associated with a decrease in TERRA expression in leukocytes. Rebound TERRA expression (returning to the level similar to the non-sarcopenic controls) was observed in the sarcopenic group after exercise and nutrition intervention. Future studies are warranted to examine the potential of TERRA as a biomarker for sarcopenia and its subsequent responses to intervention.

## 1. Introduction

The impact of sarcopenia has been well recognized in recent years, based on its association with several adverse health conditions, such as depression [1], cognitive impairment [2], a higher risk of falling, and an increased incidence of hospitalization [3]. Aging-related loss of muscle mass and function defines sarcopenia, which can result from physical inactivity, hormonal changes, and malnutrition [4]. Another contributing factor that has been highlighted recently is chronic inflammation, which leads to the release of pro-inflammatory cytokines and generation of reactive oxygen species [4]. An increase in oxidative stress causes disruption of deoxyribonucleic acid (DNA) [5], including the telomeric tract. The telomere functions as a protector against degradation at chromosomal ends [6], and contains tandem repeats of 5′-TTAGGG-3′. The association of telomere length with exercise and nutrition has been highlighted by recent studies. In 2019, the meta-analysis conducted by Lin et al. [7] found that the telomere length was longer in physically active subjects than inactive individuals, regardless of exercise intensity. In 2020, the meta-analysis done by Canudas et al. [8] demonstrated that better adherence to Mediterranean diet was associated with longer telomere length. Like sarcopenia, shortening of telomere length is an aging-related process, and their interplay remains poorly understood. Several studies [9,10,11] have been conducted to investigate whether sarcopenia is associated with differences in telomere length, but a conclusive association is awaited.

Telomere length is regulated by telomerase, which has recently been found to be associated with telomeric repeat-containing RNA (TERRA) [12]. TERRA, a long non-coding ribonucleic acid (RNA) consisting of UUAGGG repeats, is transcribed by RNA polymerase II at the subtelomeric region of a chromosome, towards the telomere [13]. It is believed that TERRA can interact directly with telomeres or attach indirectly to the related telomeric proteins to preserve the telomere’s structural stability [13]. Dysregulation or reduced production of TERRA and telomere shortening has been associated with ICF (immunodeficiency, centromeric region instability, facial anomalies) syndrome [14]. To date, no study has investigated the association of TERRA expression with aging and sarcopenia. Furthermore, as physical inactivity and malnutrition are known causes of sarcopenia, it would be of clinical merit to elucidate the mechanism by which TERRA expression changes after exercise and nutrition intervention. This study aimed to explore the impact of sarcopenia on telomere length and TERRA expression, and changes following exercise and nutrition intervention in the sarcopenic population.

## 2. Method

### 2.1. Research Participants

Older adults (age ≥ 65 years) were recruited as research participants from a geriatric clinic during the annual health check. Attendees were excluded if they were not able to ambulate without assistance, comprehend verbal commands, and respond to the questionnaire. Those with current malignancy under treatment and known, uncontrolled medical conditions (such as severe infection and unstable angina) were not included. The study was approved by the institutional review board (IRB No. 201601091RIND) of the National Taiwan University Hospital. All participants underwent measurements of grip strength and body composition, and provided written informed consent before participation in the study. Trial registration: ClinicalTrials.gov: NCT02779088. Registered 20 May 2016, https://clinicaltrials.gov/ct2/keydates/NCT02779088.

### 2.2. Anthropometric Measurement

Before the examinations, all participants were informed to undergo overnight fasting for a minimum of eight hours. They were required to undress and use gowns during the measurement. A standard digital weight and height meter was used to measure the body weight and height. Its precision was up to 100 g in weight and 1 mm in height. Body mass index (BMI) was defined as the weight divided by height squared (kg/m^2^). Whole-body dual-energy X-ray absorptiometry (DXA, Stratos dR, DMS Group, Paris, France) was used to examine body composition [15].

### 2.3. Grip Strength

Grip strength was measured using the Baseline^®^ hydraulic hand dynamometer (Fabrication Enterprises Inc., Irvington, NY, USA) over the dominant hand. The participants were invited to strenuously squeeze the device three times with an interval of 1 min between each attempt. The maximal value was used to denote grip strength [15].

### 2.4. Diagnosis of Sarcopenia

Sarcopenia was diagnosed according to the consensus of the European Working Group on Sarcopenia in Older People (EWGSOP) in which low muscle strength and low muscle mass are presented as criteria [16]. As suggested by the EWGSOP guidelines, low muscle mass was defined as a skeletal muscle mass index (SMI) of <7.40 kg/m^2^ for men and <5.14 kg/m^2^ for women. Low muscle strength was defined as a grip strength of <30 kg for men and <20 kg for women.

### 2.5. Cell Lines and Culture Conditions

HeLa (cervical cancer) and U2OS (osteosarcoma) cells were obtained from ATCC^®^. HeLa cells were cultured in the Dulbecco’s Modified Eagle medium (DMEM, Hyclone, Oslo, Norway, Cat# SH30285.02) with 10% fetal bovine serum (FBS, Thermo Fisher Scientific, Oslo, Norway, Cat# 10437-028) and 100 U/mL of penicillin-streptomycin (Thermo Fisher Scientific, Cat# 15140-122), and incubated in 5% CO_2_ at 37 °C. TERRA knockdown U2OS cells were generated using an RCas9 system [17], in which the single-guide RNA (sgRNA) sequence was replaced by TERRA antisense or control sequences. U2OS cells were transfected with Rcas9 plasmids and selected by puromycin.

### 2.6. Northern Blot Analysis

The protocol is described in a previous study [18]. Northern probes were end-labeled with isotopes using T4 polynucleotide kinase. Hybridization was carried out at 42 °C overnight using the ULTRAhyb^®^-Oligo hybridization buffer (Thermo Fisher) or Church’s buffer.

### 2.7. Measurement of Telomere Length

Blood samples were collected from the participants after at least eight hours of overnight fasting. Ten-milliliter volumes of venous blood samples were collected by venipuncture into a polypropylene tube that contained 4 mM ethylenediaminetetraacetic acid (EDTA, Sigma, Darmstadt, Germany). Cell-free plasma and buffy coats were prepared by centrifugation at 1500× *g* for 20 min at 4 °C. The samples were then stored in 500-μL aliquots at −80 °C prior to RNA extraction. Genomic DNA was extracted from frozen human buffy coats using the QIAamp^®^ DNA Mini Kit (QIAGEN, Hilden, Germany, #Cat 51306). Quantitative analysis was performed by quantitative polymerase chain reaction (qPCR) on the CFX Connect Real-Time PCR Detection System. The relative length of telomere for each participant was calculated using the telomere-to-single copy gene (T/S) ratio, defined as the number of repeats of the telomere (T) divided by a standard reference DNA (S) [19]. In the present study, the *36B4* gene located on chromosome 12 was used as the single-copy DNA. A standard curve employing genomic DNA extracted from HeLa cells was used for calibration. We conducted a series of dilutions for qPCR of both the telomere and *36B4* (*36B4* forward primer sequence: 5′-CAGCAAGTGGGAAGGTGTAATCC-3′, and *36B4* reverse primer sequence: 5′-CCCATTCTATCATCAACGGGTACAA-3′; telomeric DNA forward primer sequence: 5′-GGTTTTTGAGGGTGAGGGTGAGGGTGAGGGTGAGGGT-3′ and telomeric DNA reverse primer sequence: 5′-TCCCGACTATCCCTATCCCTATCCCTATCCCTATCCCTA-3′).

### 2.8. RNA Isolation and Quantification of TERRA

RNA was extracted from buffy coat samples using TRIzol (Thermo Fisher Scientific, Cat# 15596018) and further purified by acid-phenol:chloroform (Thermo Fisher Scientific, Cat# AM9722) reagent. Reverse transcription–quantitative polymerase chain reaction (RT-qPCR) was used for quantification of TERRA. The samples were first treated with ezDNase^™^ Enzyme (Thermo Fisher Scientific) to eliminate contaminating genomic DNA. Reverse transcription was carried out using the SuperScript^™^ IV system (Thermo Fisher Scientific, Cat# 18090200) and random (2.5 nM) hexamers combined with telomeric repeat primers (0.25 nM) to enrich TERRA complementary DNA (cDNA). The reaction was performed at 25 °C for 10 min, 50 °C for 50 min, and ended at 85 °C for 5 min. The protocol for TERRA qPCR was similar to that for the telomere qPCR. Beta-2 microglobulin (*B2M*) was used as the reference gene. TERRA expression in U2OS cells was used to obtain a standard curve for calibration between samples from different qPCR plates. The relative TERRA expression level was determined using the TERRA RNA/B2M RNA ratio.

### 2.9. Exercise and Nutritional Intervention

Older adults diagnosed with sarcopenia were invited to join a randomized controlled trial (ClinicalTrials.gov: NCT02779088) comparing the effectiveness of early versus delayed exercise and nutritional intervention on sarcopenia. Telomere length and TERRA expression were measured before and after 12 weeks of intervention. The comparison of the intervention effect on telomere length and TERRA expression was a post-hoc analysis of the aforementioned trial. The exercise protocol included a 10-min warm-up exercise; 3 sets of 10 repetitions of strengthening exercises of leg press, leg extension, and leg curl; and a 10-min cool-down exercise comprising bicycling. The frequency of exercise training was twice per week. During the intervention period, the participants were provided with nutritional supplementation. The regimen comprised two sticks of branched-chain amino acids (BCAA-Amino Vital Pro^®^, Ajinomoto, Tokyo, Japan) and two tablets of Caltrate supplement containing 600 mg calcium and 800 IU vitamin D3 (Pfizer, New York, NY, USA) every day. Each stick (3.6 g) contained 0.54 g of leucine, 0.43 g of isoleucine, 0.36 g of valine, 0.65 g of glutamine, 0.61 g of arginine, and 1.01 g of other amino acids.

### 2.10. Statistical Analysis

Continuous variables have been presented as mean ± standard deviation (SD) at 95% confidence interval (CI). The Shapiro-Wilk test was used to determine whether the variables were normally distributed. The between-group differences were analyzed by one-way analysis of variance (ANOVA) in the case of a normal distribution or the Mann-Whitney *U* test without normal distribution. Categorical data were presented as numbers and percentages and were compared using the chi-squared or the Fisher’s exact (for sparse data) tests. The impact of exercise and nutritional intervention on telomere length and TERRA expression in participants with sarcopenia, was examined using a repeated-measures ANOVA. The association of telomere length and TERRA expression with age, sex, sarcopenia, and intervention (via nutrition and exercise) was further validated by the generalized estimating equation (GEE) [20]. The GEE model is suitable for interpreting longitudinal data, such as the outcome from the sarcopenic group before and after intervention on the same person. Pearson’s correlation coefficient (r) was used to measure the correlation of telomere length and TERRA expression with grip strength and SMI. The analyses were implemented using statistical software, namely MedCalc version 14.0 (MedCalc Software, Ostend, Belgium) and SPSS (IBM SPSS Statistics for Windows, Version 21.0. Armonk, NY, USA: IBM Corp). The statistical tests were 2-tailed. Values of *p* < 0.05 were considered statistically significant.

## 3. Results

### 3.1. Basic Demographics

A total of 36 sarcopenic and 36 non-sarcopenic research participants were included in the study. There was no significant between-group difference in age, gender, and body height. The mean body weight, BMI, grip strength, and SMI values were lower in the sarcopenic participants compared to those in the controls (Table 1). 

### 3.2. Telomere Length

**Figure 1:**
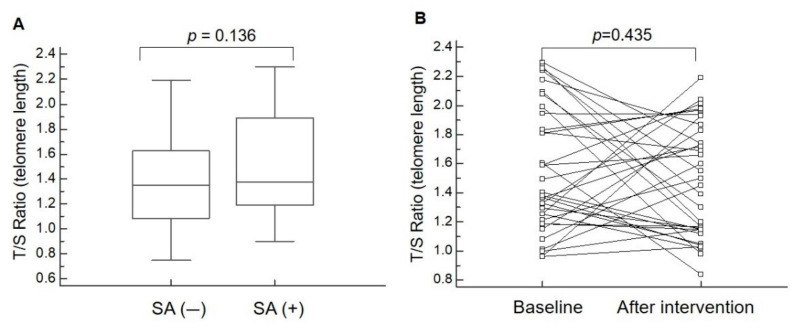
There is no significant difference in telomere length between controls and sarcopenic participants. (**A**) Comparison of the T/S ratio (telomere/single copy gene) for telomere length between research participants with and without sarcopenia (SA). (**B**) Comparison of the T/S ratio for sarcopenic participants at baseline and after exercise and nutrition intervention.

There was no significant difference in the T/S ratio between the non-sarcopenic and sarcopenic research participants (Table 1 and Figure 1A). Among the sarcopenic participants, the repeated-measured values obtained by ANOVA implied that exercise and nutrition intervention did not result in changes in the T/S ratio (Table 2 and Figure 1B).

**Figure 2:**
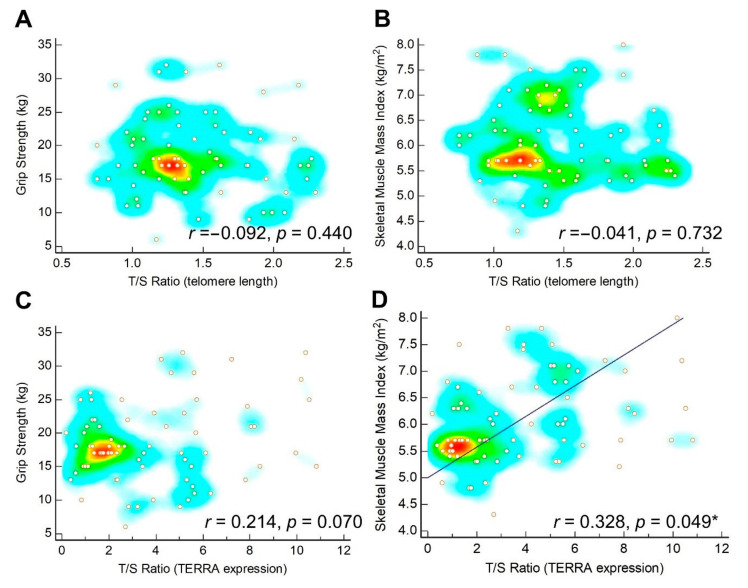
Scatter plots for correlation (**A**) between telomere length and grip strength, (**B**) between telomere length and skeletal muscle mass index, (**C**) between expression of telometric repeat–containing RNA (TERRA) and grip strength, and (**D**) between TERRA expression and skeletal muscle mass index. The regression line is plotted when *p* < 0.05. The heat map with background color coding suggests clusters of observations. * denotes *p* < 0.05.

GEE analysis demonstrated that an increase in age was negatively associated with the T/S ratio for telomere length. There was no significant association between the T/S ratio, and sarcopenia and intervention (Table 3). Finally, the correlation analysis revealed that the T/S ratio for telomere length was not correlated with grip strength or SMI (Figure 2A,B).

### 3.3. TERRA Expression

**Figure 3:**
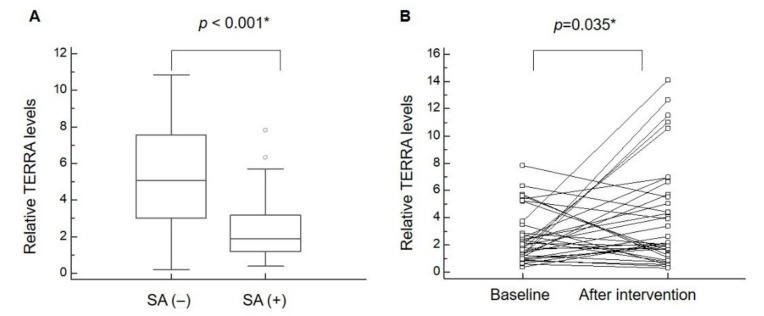
TERRA expression in leukocytes increases after exercise and nutrition intervention in participants with sarcopenia. (**A**) The expression of telomeric repeat–containing RNA (TERRA) between participants with and without sarcopenia (SA). (**B**) The expression of TERRA at baseline and after exercise combined with nutrition intervention. * denotes *p* < 0.05.

Compared to the controls, the sarcopenic research participants displayed a significant reduction (*p* < 0.001) in TERRA expression in leukocytes (Table 1 and Figure 3A). Among the sarcopenic participants, the repeated-measured values obtained by ANOVA implied that exercise and nutrition intervention resulted in a significant increase in TERRA expression (Table 2 and Figure 3B). The GEE analysis demonstrated that TERRA expression was negatively associated with sarcopenia but positively associated with nutrition and exercise intervention (Table 3). Finally, TERRA expression was likely correlated with grip strength (r = 0.214, *p* = 0.070) and was significantly associated with SMI (r = 0.328, *p* = 0.049) (Figure 2C,D).

## 4. Discussion

This study presented several novel findings. First, although the relative telomere length did not differ between participants with and without sarcopenia, a significantly lower expression of TERRA was observed in the sarcopenic group. Second, the intervention combining exercise training and nutrition supplementation had a minimal impact on telomere length but increased TERRA expression in participants with sarcopenia.

The data of the present study revealed that the T/S ratio for telomere length was negatively associated with age, but not sarcopenia. This finding was compatible with known information on shortened telomere length with aging. As sarcopenia is considered one of the most severe geriatric syndromes, several studies have been conducted to examine its association with telomere length. In 2014, Marzetti et al. included 142 older adults (aged ≥ 65 years) in a study, to measure their telomere length in peripheral blood mononuclear cells using qPCR [21]. The sarcopenic participants, determined by EWGSOP [16], had a shorter telomere length analyzed by T/S ratio, when compared to the non-sarcopenic participants. In the same year, Woo et al. investigated 2006 community-dwelling Chinese participants aged 65 years or older, in a five-year prospective cohort study [11]. Although they found that the decline in grip strength was less significant in those in the highest quartile of telomere length (also quantified by the T/S ratio) than in the lower quartiles, sarcopenia, defined by the Asian Working Group for Sarcopenia (AWGS) algorithm [22], was not associated with telomere length. In 2018, Rippberger et al. enrolled 2672 participants aged ≥ 60 years to estimate their telomere length using a method similar to the aforementioned two studies [10]. They demonstrated a potentially inverse relationship between telomere length and mortality in those without sarcopenia but not in the sarcopenic population, diagnosed by the criteria of the Foundation for the National Institutes of Health (FNIH) [23]. In our study, we also noticed a lack of association between sarcopenia and telomere length. Although sarcopenia and shortened telomere length share certain common mechanisms, such as chronic inflammation and increased oxidative stress, the telomere length is unlikely to act as an exclusive biological indicator of sarcopenia.

We found that the T/S ratio for the telomere did not change after exercise training and nutritional supplementation in the sarcopenic participants. A recent review indicated inconsistent effects of physical activities on the telomere length of skeletal muscles and leukocytes, based on observational studies [24]. Evidence from interventional trials revealed no significant differences in leukocyte telomere length between those receiving aerobic training and those that did not [24]. In our study, the telomere length of the sarcopenic participants at baseline was similar to that of the controls. Therefore, even if exercise and nutritional support did have some influence, the effect would be difficult to recognize on telomeres with age-matched normal length.

Our results revealed that TERRA expression was higher in non-sarcopenic participants than that in sarcopenic participants. TERRA plays a crucial role in the modulation of telomere length in a telomere length-dependent manner. For cells with longer telomeres, TERRA is likely to compete with telomerase’s DNA substrate or enhance the catalytic reverse transcriptase subunit of telomerase, both inhibiting the elongation of telomeric repeats [14]. In contrast, for cells with shorter telomeres, TERRA may promote telomere lengthening by facilitating the recruitment of telomerase. In 2013, Cusanelli et al. reported that TERRA expression was stimulated by telomere shortening in yeast cells and further expedited the formation of TERRA-telomerase clusters for the preparation of telomere elongation [25]. Therefore, the increased TERRA expression in healthy older adults may act as a normal cellular response to protect the telomere from attrition.

**Figure 4:**
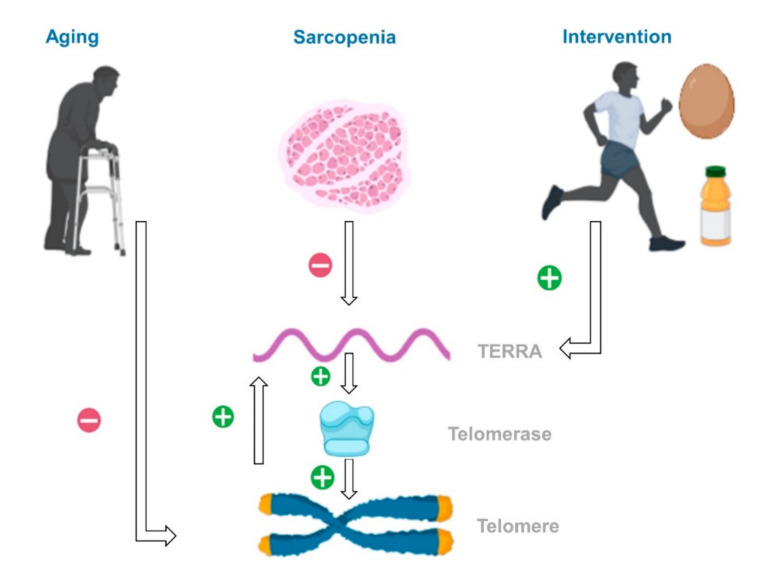
A plausible pathway illustrating how aging, sarcopenia, and intervention using exercise and nutrition affect the telomere and telomeric repeat-containing RNA (TERRA).

We proposed two possible pathways to link decreased TERRA expression and sarcopenia (Figure 4). First, the promoter of TERRA includes abundant cytosine-phosphate-guanine (CpG) sites, which are targets for DNA methyltransferases [26]. A previous cross-sectional study demonstrated significantly less total methylation of differentially methylated CpG sites in sarcopenic women than that in non-sarcopenic controls [27]. Differences in DNA methylation might play a role in the down-regulation of TERRA expression. Second, the regulation of TERRA may be mediated through heterogeneous nuclear ribonucleoprotein (hnRNP), which attaches to various parts of TERRA [26]. Abnormal distribution of hnRNP in muscle biopsy specimens has been found in certain neuromuscular diseases, such as inclusion body myositis [27]. TERRA expression may be affected in patients with sarcopenia through hnRNP, which plays an important role in skeletal muscle metabolism and growth.

An increase in TERRA expression was observed in our sarcopenic participants, after exercise and nutritional support. Several previous meta-analyses have proven benefits of exercise and nutritional intervention for patients with sarcopenia, including an increase in lean body mass, walking speed, grip strength, knee extensor power, and a decline in fat mass [28,29]. The association of exercise and nutrition with epigenetic regulation has been uncovered in recent years. Exercise is known to affect DNA methylation, histone modification, and microRNA expression in muscle cells [30]. Furthermore, exercise has been demonstrated to elicit noticeable physiological and cellular adaptations in skeletal muscles and to achieve a new positive balance between antioxidant defense mechanisms and reactive oxygen species [31]. In terms of nutrition intervention, amino acids play a role in the phosphorylation of translational factors critical for muscle hypertrophy [32]. Micronutrients, such as vitamin D, are also involved in the regulation of methylation of certain genes [33]. Therefore, although the exact pathway for rebounding TERRA expression after exercise and nutritional intervention is unknown, we suggest that this change may occur at the epigenetic level of gene regulation.

Through the GEE analysis, we could not identify a sex-specific difference in telomere length and TERRA expression. Rippberger et al. investigated 2672 older adults and found that men tended to have shorter telomeres compared to that in women in the sarcopenic population [10]. However, Saum et al. included 3537 community-dwelling adults aged 50 to 75 years in a study and found that the relative telomere length defined by the T/S ratio was not related to frailty in different sex subgroups [34]. Estrogen has been shown to upregulate telomerase and exerts a protective effect on telomeres against reactive oxygen species [35]. It is reasonable to contemplate the potential influence of gender on telomere length and TERRA expression. In our study, as sex was included in the GEE model, we could assert that the association of telomere length and TERRA expression with sarcopenia and intervention using exercise and nutrition was independent of gender.

**Figure 5:**
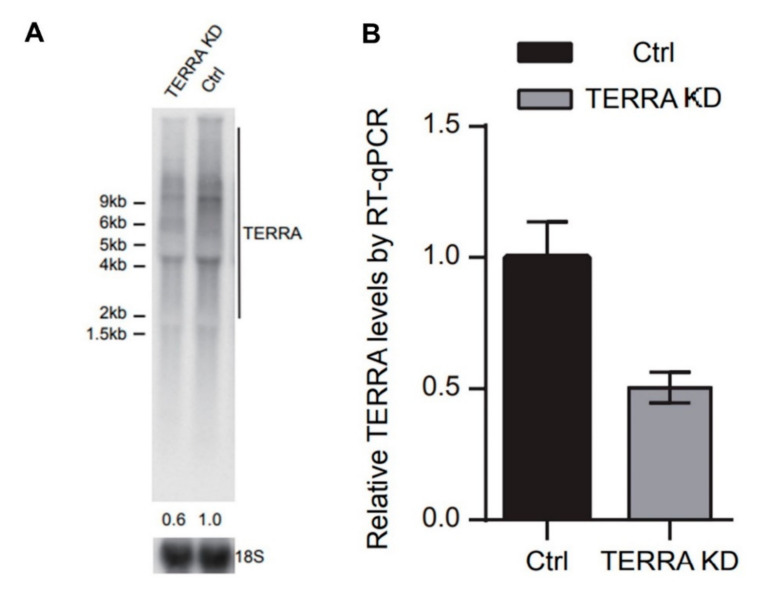
TERRA level detected by RT-qPCR is comparable with the results of northern blot analysis. (**A**) Northern blot analysis for detection of TERRA RNA level in U2OS cells after TERRA knockdown (KD). The TERRA levels were normalized with 18S rRNA expression levels. (**B**) RT-qPCR analysis for detection of TERRA level in U2OS cells after TERRA knockdown. The TERRA levels were normalized with beta-2 microglobulin (B2M) RNA expression levels.

In the study, we tested several internal controls, such as 18s, GAPDH and beta-actin. However, the mean Cq (quantitation cycle) values of qPCR were fluctuated a lot between individuals using those primer sets. Only B2M primers routinely produced lower Cq values, thus we chose B2M as an internal control. Several papers also suggested that B2M is relatively a good reference gene compared to other controls using hematopoietic stem cells or blood cells [36,37]. Initially, we tried to use subtelomeric primer sets [38,39] to detect TERRA transcripts derived from individual chromosomes (such as 2q, 9q, XqYq), but the RNA levels from buffy coat samples were too low to obtain qualified data. Due to the limited samples from buffy coats, the quantification was only conducted by using the primers for telomeric repeats (Table S1). In order to see whether this RT-qPCR method accurately represents TERRA levels, we compared the northern blot and RT-qPCR (Figure 5) and the results were similar.

There was a lack of serial evaluations after intervention in the sarcopenic group. This is a major limitation of our study. Future studies are needed to explore the duration of the increased TERRA expression after discontinuing exercise training and nutrition supplementation. Second, there was no specific inquiry of physical activity habits of the enrolled participants. The heterogeneity of physical activity levels could be a potential confounder of telomere length and TERRA expression. Measurements of physical activity level using a validated questionnaire should be incorporated in the subsequent studies. Third, in the present study, the telomere length and TERRA expression were both measured in leukocytes. However, sarcopenia is a clinical condition that primarily affects the skeletal muscle tissue. Several previous studies [40,41] have demonstrated a positive correlation between telomere length in leukocytes and that in human skeletal muscles. Hiam et al. [41] also pointed out that the influence of aging on telomere length was different in leukocytes from human skeletal muscles. Therefore, a prospective study is required to investigate whether the telomere length and TERRA expression in skeletal muscle tissue differ from those in leukocytes in participants with sarcopenia.

## 5. Conclusions

Sarcopenia is associated with a decrease in TERRA expression. Rebound TERRA expression can be observed in sarcopenic older adults, after exercise and nutrition intervention. Future prospective studies are warranted to examine the potential of TERRA as a biomarker for sarcopenia assessment, and its subsequent response to exercise training and nutrition supplementation.

## Figures and Tables

**Table 1:** Demographics, physical performance, body composition and T/S ratio for telomere length and TERRA expression the study participants.

	Sarcopenia (−) (*N* = 36)	Sarcopenia (+) (*N* = 36)	*p* Value
Demographics and anthropometrics measurements			
Age (year)	75.69 ± 5.50 (73.83 to 77.55)	74.86 ± 6.67 (72.60 to 77.11)	0.565
Female gender (number, %)	28, 77.77%	28, 77.77%	1.000
Height (cm)	155.09 ± 7.62 (152.51 to 157.66)	154.51 ± 7.18 (152.08 to 156.95)	0.744
Weight (kg)	61.82 ± 9.76 (58.52 to 65.12)	51.39 ± 7.97 (48.69 to 54.09)	<0.001 *
Body mass index (kg/m^2^)	25.64 ± 2.88 (24.66 to 26.61)	21.45 ± 2.31 (20.67 to 22.23)	<0.001 *
Physical performance			
Handgrip strength (kg)	19.86 ± 6.52 (17.65 to 22.06)	16.91 ± 4.96 (15.23 to 18.59)	0.035 *
Body Composition			
Skeletal muscle index (kg/m^2^)	6.63 ± 0.70 (6.39 to 6.86)	5.57 ± 0.52 (5.39 to 5.75)	<0.001 *
Laboratory Measurement			
Relative telomere length	1.38 ± 0.37 (1.25 to 1.50)	1.52 ± 0.43 (1.37 to 1.67)	0.136
TERRA expression	5.18 ± 2.98 (4.17 to 6.19)	2.51 ± 1.89 (1.86 to 3.15)	<0.001 *

Values are given as mean ± standard deviation and 95% confidence interval. *p* values pertain to between-group comparisons. * indicates *p* < 0.05. TERRA: Telomeric repeat-containing *RNA*.

**Table 2:** T/S ratio for telomere length and TERRA expression of the study participants before and after exercise and nutrition intervention.

	Before Intervention (*N* = 36)	After Intervention (*N* = 36)	*p* Value
T/S ratio (telomere length)	1.55 ± 0.44 (1.40 to 1.70)	1.48 ± 0.39 (1.34 to 1.61)	0.435
T/S ratio (TERRA expression)	2.53 ± 1.94 (1.87 to 3.19)	4.09 ± 3.87 (2.77 to 5.40)	0.035 *

Values are given as mean ± standard deviation and 95% confidence interval. *p* values pertain to repeated measurement comparisons. * indicates *p* < 0.05. TERRA: Telomeric repeat-containing *RNA.*

**Table 3:** Association of the T/S ratio of the telomere length and TERRA expression with old age (≥65 years), sarcopenia and intervention using exercise and nutrition support.

	Age (Year)	Sarcopenia	Intervention	Female Gender
Telomere length	−0.017 (−0.02 to −0.005)	−0.131 (−0.31 to 0.04)	0.048 (−0.13 to 0.22)	−0.020 (−0.20 to 0.16)
	*p* = 0.004 *	*p* = 0.154	*p* = 0.605	*p* = 0.840
TERRA expression	−0.034 (−0.13 to 0.06)	−2.705 (−3.85 to −1.55)	1.599 (0.22 to 2.97)	−0.632 (−1.84 to 0.57)
	*p* = 0.492	*p* < 0.001 *	*p* = 0.023 *	*p* = 0.306

* indicates *p* < 0.05. TERRA: telomeric repeat-containing *RNA.*

## Supplementary Materials

The following are available online at https://www.mdpi.com/2072-6643/12/12/3766/s1, Table S1: List of the primers for RT-qPCR.
