# Supplementary material for: Expression of Telomeric Repeat–Containing RNA Decreases in Sarcopenia and Increases after Exercise and Nutrition Intervention
Source: Nutrients. 2020 Dec 8;12(12):3766. doi: 10.3390/nu12123766 (PMC7762552; doi:10.3390/nu12123766)
Supplement: Supplementary file 1 [file nutrients-12-03766-s001.pdf]

Supplementary Table 1. List of the primers for RT-qPCR

Primer Name	Forward Sequence	Reverse Sequence
TERRA	5’-GGTTTTTGAGGGTGAGGGTGA GGGTGAGGGTGAGGGT-3’	5’-TCCCGACTATCCCTATCCC TATCCCTATCCCTATCCCTA-3’
B2M	5’-CTATCCAGCGTACTCCAAAG-3’	5’-GAAAGACCAGTCCTTGCTGA-3’

Abbreviation: TERRA, Telomeric repeat-containing RNA. B2M, Beta-2 microglobulin
